# Silver Nanoparticle Incorporated Human Amniotic Membrane Gel Accelerates Second-Degree Burn Wound Healing in Wister Rat

**DOI:** 10.1155/2023/9808556

**Published:** 2023-04-14

**Authors:** Israt Jahan Jhumi, Tusher-Al- Arafat, Polash Chandra Karmakar, Md. Arifuzzaman, Md. Sharif Hossain, Naznin Akhtar, S. M. Asaduzzaman

**Affiliations:** ^1^Department of Biotechnology & Genetic Engineering, Jahangirnagar University, Savar, Dhaka 1342, Bangladesh; ^2^Institute of Tissue Banking and Biomaterial Research, Atomic Energy Research Establishment (AERE), Savar, Dhaka 1349, Bangladesh

## Abstract

Burn has terrible consequences for the affected patients, making them vulnerable to wound infections and septicemia, which results in physical and mental disability and death, necessitating superior treatment options. Human amniotic membrane (HAM) has been utilized in burn wounds for decades for its low immunogenicity, angiogenic, anti-inflammatory, and antimicrobial properties and for promoting epithelialization. Silver nanoparticles (AgNPs), on the other hand, have antimicrobial properties and promote fibroblast migration. This study aimed to determine the burn wound healing potential of HAM + AgNPs. The gel was prepared using HAM (1% and 2%), AgNPs, carbopol 934, acrylic acid, glycerine, and triethanolamine, and different physical properties (pH, water absorption, swelling variation, spreadability, etc.) of the gel were determined; nuclear magnetic resonance (NMR) spectroscopy, antibacterial activity, brine shrimp lethality test, and histopathological observation were conducted. *In vivo* studies with Wistar rats demonstrated better healing capabilities than individual components of the gel. Wound contraction percentage after 20 days was 96.1 ± 0.27% which was highly significant (*p* < 0.0001), and the epithelialization period was 23.67 ± 2.05 days (*p* < 0.01) for HAM + AgNPs which was preferable to the positive control, AgNPs, HAM, and negative control; also, the histopathologic observation using hematoxylin and eosin, and Masson's trichrome staining were showed the better healing progress for HAM + AgNPs. Both HAM and AgNPs had antibacterial activities against gram-positive and gram-negative bacteria. These results indicated that the formulated HAM + AgNPs gel had remarkable effectiveness in burn wound healing compared to others. Further studies will be conducted to determine the molecular mechanism behind wound healing.

## 1. Introduction

Burn is a destructive emergency that constitutes a major public health issue worldwide with many physical and mental disabilities [1]. Burn wound healing relies on several factors, for instance, degree of burn (I to IV), cause (e.g., thermal, electrical, radiation or chemical), quality, or health condition of the patient (immunosuppressive, acidosis, lethargic), and related comorbidities [2]. The basic principles of burn wound healing are tissue damage minimization, scab formation, would debridement, maximizing tissue perfusion as well as oxygenation. This process consists of highly connected overlapping phases including inflammation, proliferation, and remodeling [3].

The inflammatory phase carries neutrophils and monocytes to the injury site through vasodilation and extravasation of fluids triggering an immune response that is strengthened by the macrophages. Overlapping with the inflammatory phase, the proliferative phase begins with the activation of keratinocytes and fibroblasts [4, 5]. This phase plays an essential role in healing by wound closure and reconstructing the vascular network. Overlapping with the proliferative phase, remodeling is the final phase of healing where wound scar matures by deposition of collagen and elastin, and fibroblasts become myofibroblasts [4, 6].

Skin acts as the first line of defense against microorganisms and damage to skin results in loss of its defensive function which leads to a greater risk of infection causing major difficulty in burn wounds where bacteria impede the wound healing and are responsible for the deaths of the majority of burn patients [7, 8]. Bacteria are often isolated from burn wounds including *Staphylococcus aureus, Streptococcus pyogenes, Klebsiella pneumoniae, E. coli, Pseudomonas aeruginosa,* and fungi such as *Candida spp., Aspergillus Niger,* and *Zygomycetes* [9, 10]. The wound healing process is hindered by these microorganisms because they compete with host cells for nutrients and oxygen and also produce toxic waste products [11]. Additionally, spreading antibiotic resistance places burn patients at higher risk for infections which may be life-threatening [12, 13]. Thus, a product that improves wound healing as well as possesses antimicrobial activities can be more efficient in burn treatment.

In recent years, human amniotic membrane (HAM) is increasingly being used as a wound dressing in burn wounds and ophthalmology for its anti-inflammatory, low antigenic, feasibility, and cost-effectiveness [14, 15]. Additionally, its features are similar to human skin as it originates from ectoderm, and hence, it could prevent water and heat loss from the wound and impede bacterial contamination [16]. Besides, HAM as a biological dressing is superior to other allograft and xenograft for its ability to make comfort from pain, induce reepithelialization, and reduce loss of heat, protein, and energy, and this might be a superior wound dressing [17]. A recent study by Gholipourmalekabadi et al. revealed that neither fresh nor acellular HAM was able to inhibit the growth of some antibiotic-resistant bacteria isolated from burn patients [18]. Therefore, additional material is required to complement HAM grafting as an efficient dressing with the purpose of full protection of burn wounds against healthcare-associated infections (HAI). Nowadays, silver nanoparticle (AgNPs) has been promoted in wound healing based on regulating the cytokines that are involved in burn infections [19]. Liao et al. observed that AgNPs could induce the apoptosis-like reactive oxygen species (ROS) pathway of multidrug-resistant*P. aeruginosa* isolated from burn patients by elevating the levels of superoxide dismutase, catalase, and peroxidase which could result in impaired DNA and ribosome, protein degradation and declined synthesis of the macromolecules, and all the above events may work together toward the bacterial death [20]. A recent study by Wasef et al. described that AgNPs enhance healing processes in the murine burn model by modifying leukocytic infiltration and collagen degeneration [21]. Furthermore, AgNPs loaded collagen/chitosan scaffolds stimulated wound healing by regulating pro-inflammatory and scar-related factors as well as *α*-smooth muscle actin (SMA) [22].

In our study, we used human amniotic membrane-based gel embedded with AgNPs to treat burn wounds, and our study aims at determining the combined effect of HAM and AgNPs on burn wound healing.

## 2. Methods

### 2.1. Ethical Approval

The “Ethical Review Committee” of Jahangirnagar University, Savar, Dhaka, Bangladesh, has provided the ethical permission [Ref No. BBEC, JU/M 2020(12)3] for the study, and the use of human amniotic membrane for the experimental purpose was approved by the ethical committee of Atomic Energy Research Establishment and authorized by the Government of Bangladesh under the “Human Organ/Tissue Donation and Transplantation Act, 1999.” Written consent for the use of HAM for research purposes was taken from the donors.

### 2.2. Preparation of HAM + AgNPs Gels

**Figure 1:**
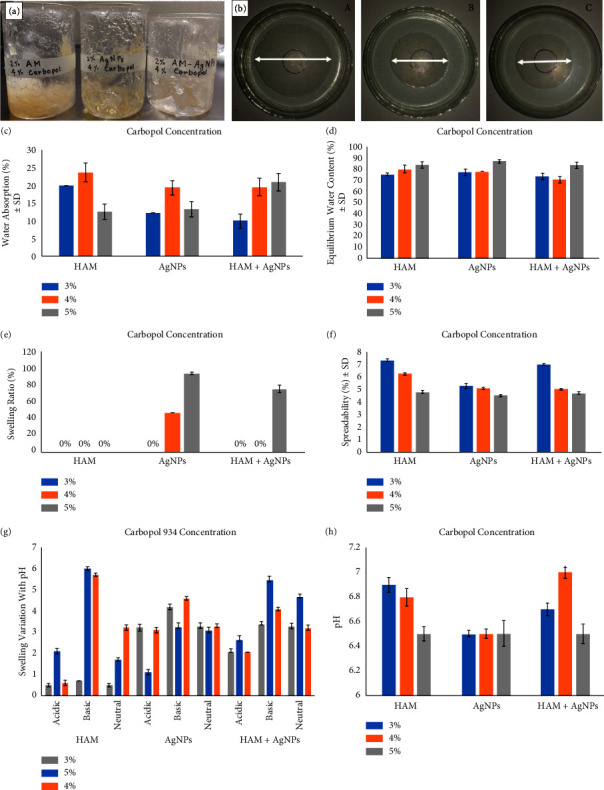
Physical appearance and physical properties of gels at different carbopol concentrations: (a) formulated gels, (b) spreadability of HAM + AgNPs gels with 3% carbopol (A), 4% carbopol (B), and 5% carbopol (C). The graph represents (c) water absorption (%), (d) equilibrium water content (%), (e) swelling ratio (%), (f) spreadability (cm), (g) swelling variation, and (h) pH.

A total of nine types of gels were prepared using HAM, carbopol, glycerine (Merck, India), acrylic acid (Sigma Aldrich, Germany), silver nanoparticles, and triethanolamine (Hi Media, India) by the previously reported method [23] with slight modification. Firstly, weighed carbopol 934 was gradually added with distilled water in a beaker and dispersed with the help of a magnetic stirrer. An accurate amount of HAM was dissolved and mixed under continuous stirring in another beaker with distilled water and homogenized using a homogenizer, and acrylic acid was added and mixed well. Then, glycerin was added slowly as a moisturizer under continuous stirring. After that, considering dose-dependent cytotoxicity, the silver nanoparticle (50 *μ*g/ml) was mixed with the solution [12]. HAM solution was then put into a beaker containing solution carbopol 934 and mixed well. Finally, to neutralize the mixture, triethanolamine was added drop by drop, and mixing was continued until a translucent gel was created (Figure 1(a)). The preparation of human amniotic membrane extract and synthesis of AgNPs have been mentioned in the supplementary information (SI) files.

### 2.3. Characterization of the Formulated Gels

Characterization of the formulated gels has been mentioned in the supplementary information (SI) files.

#### 2.3.1. Determination of Physical Properties of Gels

Water absorption, swelling ratio, equilibrium water content, and swelling variation with pH determination have been performed (Figures 1(b)–1(h)). Details of the procedures have been mentioned in SI files.

#### 2.3.2. Nuclear Magnetic Resonance (NMR) Spectroscopy

The formulated gels are analyzed using NMR (AscendTM 400, BRUKER) in the liquid state at Wazed Miah Science Research Center Jahangirnagar University, Savar, Dhaka-1342. The experiment was operated at 11.7 T, 400 MHz for 1H, no of scan 128, and acquisition time 2.7 sec, and chloroform is used as the solvent.

### 2.4. Determination of Antibacterial Activity by Disc Diffusion Method

Antimicrobial susceptibility testing was performed by the disc diffusion method to evaluate the presence of antibacterial activities of the formulated gel according to the standard method as described earlier [9, 23]. The antibacterial activity of three types of gels was evaluated against *Staphylococcus aureus* (ATCC 25923), *Klebsiella pneumoniae* (ATCC 70084), *Pseudomonas aeruginosa* (ATCC 27853), and *Escherichia coli* (ATCC 25922), and the commercial antibiotic disk was used as +Ve control. The formulated gels were plated in the Petri dishes containing bacterial lawn, the plates were examined for the inhibition zone after 24 hrs of incubation at 37°C, and each assay was performed thrice.

### 2.5. Quantitative Analysis of the Antibacterial Activity

Bacterial colonies of *Staphylococcus aureus* (ATCC 25923), *Klebsiella pneumoniae* (ATCC 70084), *Pseudomonas aeruginosa* (ATCC 27853), and *Escherichia coli* (ATCC 25922) were picked from Mueller Hinton agar (MHA) and suspended in Mueller Hinton broth. The cultures were grown aerobically for 20 h with continuously shaken at 100 rpm at 37°C. For antibacterial activity assays, 1 mL of each culture was diluted with MHB medium to an optical density (OD) of 0.1 at 600 nm (OD, 0.1 represents 10^8^ cells/ml) using a spectrophotometer. Then, formulated gels (0.1 mg) were added to the MHB broth (5 ml) containing different bacteria, and the growth of the bacteria was measured at 0 hr, 1 hr, 2 hr, 3 hr, 4 hr, 5 hr, and 6 hr using a spectrophotometer [24, 25].

### 2.6. Brine Shrimp Lethality Test

The brine shrimp lethality test is a simple and efficient cytotoxicity test of bioactive materials depending on the killing capability of the sample on brine shrimp (*Artemia Salina*) [26, 27]. The procedure of the brine shrimp lethality test has been mentioned in the SI files.

### 2.7. In Vivo Study

Female Wistar rats were collected from the Department of Pharmacy, Jahangirnagar University, Savar, seven days before the experiment for acclimatization with the environment. Rats were fed with a standard diet prepared in the Laboratory of the Department of Biochemistry and Molecular Biology, Jahangirnagar University, Savar. Female Wistar rats weighing 117 ± 25 g were taken and randomly divided into five groups (*n* = 3). Water and food intake, and body weight measurement of the rats were taken every day during the experiment conducted (SI Figure 1).

#### 2.7.1. Skin Irritation Study

The dorsal hairs of six female Wistar rats (*n* = 2) were shaved on the day of the experiment and grouped randomly. The gels containing HAM, AgNPs, and HAM + AgNPs were applied on animals daily for up to 7 days, and lastly, the treated skins were observed for erythema and oedema (Figure 5(a)).

#### 2.7.2. Creation of Wound and Dosing Schedule

Wounds were created by heated aluminum discs kept in a water bath for 30 minutes at 100°C. For the determination of wound healing activity of the different formulated gels, 1 g of each gel was applied twice daily for 20 days to treat burn wound of different rat groups (*n* = 3) except positive control where commercial burn cream (silver sulfadiazine) was applied and negative control where no gel or cream was applied.

#### 2.7.3. Percentage Wound Contraction and Epithelialization Period

The area of the wound was expressed as a percent to measure the wound contraction. From this, wound areas were calculated on respective days, and the wound contraction percentage was calculated by taking the initial size of the wound (491 mm^2^) as 100% [23].

Percentage wound contraction = [(initial wound area − final wound area)/initial wound area] × 100.

Percentage wound contraction and epithelialization period have been mentioned in SI files.

#### 2.7.4. Histopathological Observation

The regenerating skin tissues of the rats around the wounds were collected individually on days 0, 7, 14, and 21. For observing the wound healing progress through histopathologic assessment, hematoxylin and eosin (H&E) and Masson's trichrome staining were conducted [22, 28]. After being fixed in paraformaldehyde (4%) for 24 h, the samples were sectioned at 4 *μ*m by a microtome (Ogawa Seiki Co; Ltd, Japan). Then, H&E and MT staining were conducted, and images were captured using light microscopy (Leica ICC50 E, Germany) [29].

### 2.8. Statistical Analysis

Results were expressed as mean ± standard deviation (SD) and SD, as well as *p* values, and were calculated by Student's *t*-test using SPSS. The significant values were considered as ^∗^*p* ≤ 0.05, ^*∗∗*^*p* ≤ 0.01, ^*∗∗∗*^*p* ≤ 0.001, ^*∗∗∗∗*^*p* ≤ 0.0001.

## 3. Results

### 3.1. Physical Characteristics of the Formulated Gels

All gel formulations were analyzed based on physical characteristics to identify the best gel with different carbopol 934 concentrations (Figures 1(a)–1(h)). The pH of all formulations was found between 6.5 and 7.0 which denotes that those gels were suitable to use on the skin.

### 3.2. Evaluation of Formulated Gels

#### 3.2.1. Physical Appearance, Homogeneity, and Spreadability of the Formulated Gels

All gels were homogeneous and have a good consistency. The color of the HAM gel was off-white while the gel containing both HAM and AgNPs was whitish yellow, and AgNPs gel was light yellow (Figure 1(a)). Spreadability showed a variation with carbopol concentration. Highly concentrated carbopol gels tend to be much thicker and spread less. The spreadability of 3% carbopol gel was greater than both 4% and 5% carbopol gel (Figure 1(b)).

### 3.3. Selection of Optimum Gel

After observing the physical properties of formulated gels including water absorption, swelling variation with pH, equilibrium water content, and swelling ratio, HAM + AgNPs gel with 4% carbopol expressed better physical characteristics than other gel formulations (Figures 1(b)–1(h)). In the case of spreadability, lower spreadability values of 5% carbopol gel tend to be thicker and spread less, and higher spreadability values of 3% carbopol gels indicate the lower thickness and spread more. The concentration of 4% carbopol gels remains optimum and appropriate gel texture, and thickness can be maintained.

### 3.4. Nuclear Magnetic Resonance (NMR) Spectroscopy

**Figure 2:**
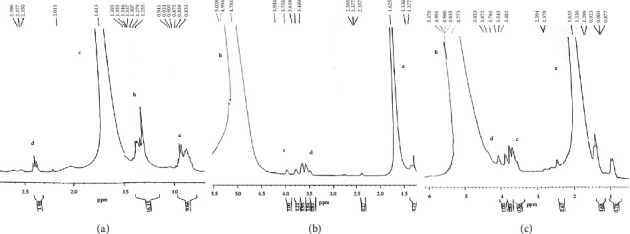
NMR spectroscopy of the formulated gels. (a) 2% HAM and 4% carbopol, (b) 2% AgNPs and 4% carbopol, and (c) 2% HAM + AgNPs and 4% carbopol.

HAM, AgNPs, and HAM + AgNPs gels were characterized using NMR spectroscopy. In the NMR analyses, the demand temperature on the temperature unit (TE) was 298.2 K, the nucleus for channel f1 (NUC 1) was 1H, the irradiation frequency for channel 1 was 400 MHz, the number of scan (NS) was 128, and dwell time was 41 *μ*s. The ^1^H NMR spectrum using chloroform solvent (CDCl3) of the formulated HAM gel contains signals where chemical shift (*δ*) at ppm 0.9, multiple (m) signals, which resemble alkyl (methyl) group (a), chemical shift (*δ*) at ppm 1.3, multiple (m) signals which represent alkyl (methylene) group (b), chemical shift (*δ*) at ppm 2.0, singlet (s) represents hydroxyl group (c) of human amniotic membrane, chemical shift (*δ*) at position of 2.38 ppm and 2.35 ppm, triplet(t) denotes carbopol 934 (d) present in the gel which is shown in Figure 2(a). The ^1^H NMR(CDCl3) spectrum of AgNPs gel contains signals where chemical shift (*δ*) at ppm 1.62(s) which represents alkyl (methine) group (a), chemical shift (*δ*) at ppm 4.9(s) represents amine (b), chemical shift (*δ*) at a position of 3.55 ppm and 3.61 ppm (m) denotes glycerol (d), and chemical shift (*δ*) at position of 2.37 ppm and 2.35 ppm, triplet(t) denotes carbopol 934 present in the gel which is shown in Figure 2(b). The ^1^H NMR(CDCl3) of HAM + AgNPs gels contains multiple signals where chemical shift (*δ*) at ppm 1.63 (s) which denotes alkyl (Methine) group (a), chemical shift (*δ*) at ppm 4.9, singlet denotes amine groups (d) of amniotic membrane. Chemical shift (*δ*) at a position of 3.55 ppm and 3.62 ppm (m) denote glycerol, chemical shift (*δ*) at 3.60 ppm (*t*) represents trimethyl amine, and at 3.9 ppm (m) represents acrylic acid (d) presents in the gel (Figure 2(c)).

NMR spectroscopy of the other gels (2% HAM and 5% carbopol; 2% AgNPs and 5% carbopol; 2% HAM + AgNPs and 5% carbopol) has been mentioned in Supplementary Figure 2.

### 3.5. Antimicrobial Activity

**Figure 3:**
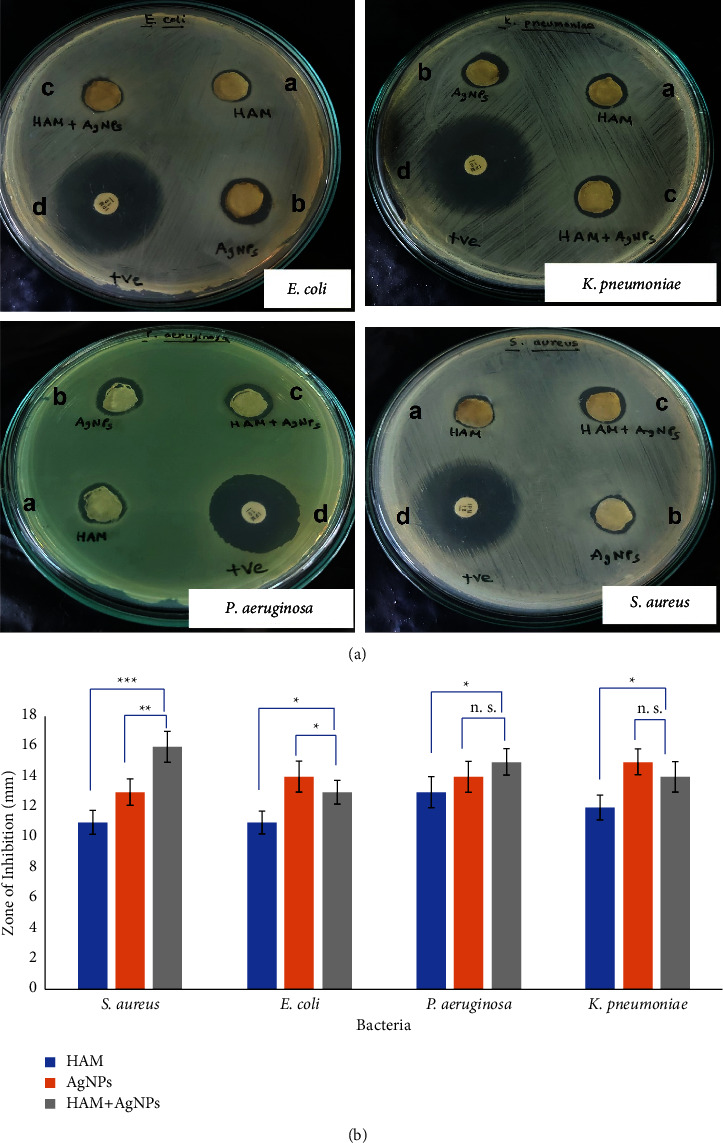
Antimicrobial activity of the formulated gels. (a) Antimicrobial activity of the gels: HAM(A), AgNPs(B), HAM + AgNPs(C), and +ve control (D) against *Staphylococcus aureus, Escherichia coli, Pseudomonas aeruginosa*, and *Klebsiella pneumoniae*. (b) Bar diagram of the zone of inhibition in the antimicrobial study (level of significant was ^*∗*^*p* ≤ 0.05, ^*∗∗*^*p* ≤ 0.01, ^*∗∗∗*^*p* ≤ 0.001).

The antimicrobial activities of the selected gels against the bacterial strains were assessed by the presence of inhibition zones. The gels showed antibacterial activities against *S. aureus*, *K. pneumoniae*, *P. aeruginosa*, and *E. coli* (Figure 3(a)). HAM + AgNPs have shown significant (^*∗*^*p* ≤ 0.05) zone of inhibition compare to HAM against *S. aureus, E. coli, and P. aeruginosa*. On the other hand, HAM + AgNPs have shown significant (^*∗*^*p* ≤ 0.05) zone of inhibition against *P. aeruginosa* and *K. pneumoniae* in comparison with AgNPs (Figure 3(b)).

### 3.6. Quantitative Analysis of the Antibacterial Activity

**Figure 4:**
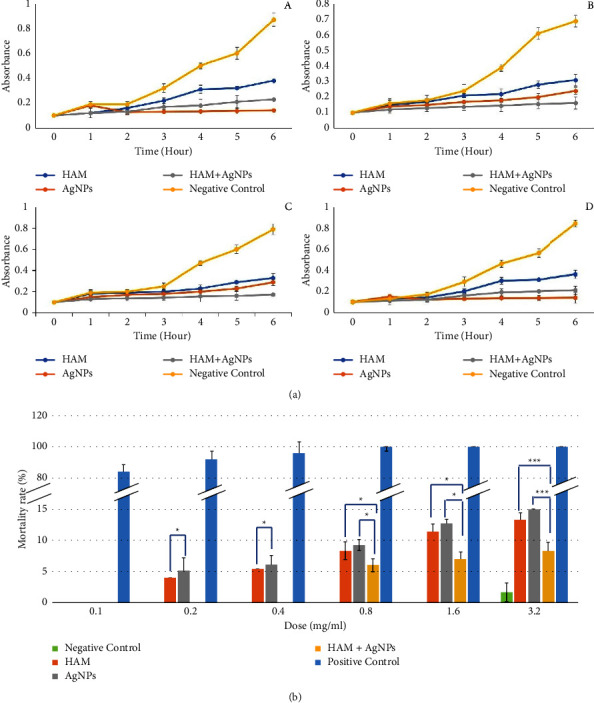
Quantitative analysis of the antibacterial activity of the gels and Brine shrimp lethality test. (a) Antimicrobial activity of the gels against *Escherichia coli* (A), *Staphylococcus aureus* (B)*, Pseudomonas aeruginosa* (C), and *Klebsiella pneumoniae* (D). (b) Brine shrimp lethality test of the gel; mortality rate of brine shrimps at different concentrations of formulated gel (the level of significance was ^*∗*^*p* ≤ 0.05, ^*∗∗*^*p* ≤ 0.01, ^*∗∗∗*^*p* ≤ 0.001).

The bacterial growth inhibition was measured by the optical density (OD) of the bacterial culture containing the formulated gels at 0 hr, 1 hr, 2 hr, 3 hr, 4 hr, 5 hr, and 6 hr. Compared with the negative control (no sample), the optical. HAM + AgNPs showed greater growth inhibition against *Staphylococcus aureus* and *Pseudomonas aeruginosa,* whereas AgNPs showed higher inhibition against *Escherichia coli* and *Klebsiella pneumoniae* (Figure 4(a)).

### 3.7. In Vitro Cytotoxicity Test

The results of the lethality test expressed that, in the case of higher doses of gel samples, there was an increased rate of death of nauplii and lower doses of gels which showed very little or no cytotoxic effect as the death rate was minimal. HAM gel and AgNPs gel expressed a little cytotoxicity at higher doses, but HAM + AgNPs gel expressed no cytotoxicity at lower doses and minimal cytotoxicity at higher doses (Figure 4(b)).

### 3.8. Skin Irritation Studies

**Figure 5:**
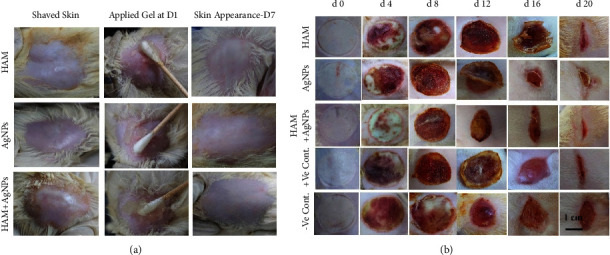
Skin irritation study and macroscopic observation of wound. (a) Skin irritation study on shaved skin: topical application of gel at day 1 and skin appearance at day 7 (no oedema and erythema on skin after 7 days) and (b) macroscopic observation of wound after applying different types of gels at day 0, 4, 8, 12, 16, and 20.

After the application of selected gels to the rats for 7 days, there was no induction of any oedema or erythema (Figure 5(a)) which expressed the gel's applicability and safety.

### 3.9. Physiological Properties of Rats

A study of physiological conditions of different rat groups concluded that the body weight of rats of control groups (positive and negative group) was increased slightly, while the body weight of HAM + AgNPs gel treated rats was increased more than HAM and AgNPs gel treated rats (SI Figure 1). However, the amount of average food intake was highest in HAM-treated rats, and average water intake was highest in HAM + AgNPs (SI Figure 1). The results showed that all rats had moderate body weight as well as food and water intake which indicated that all rats were healthy during the treatment days.

### 3.10. Wound Treatment and Gel Application

The representative images of wounds and treatment for each of the time points (day 0, 4, 8, 12, 16, and 20) are shown in Figure 5(b). Second-degree burn produced a burning injury that was white in color and hyperemia occurred subsequently in the wound area. The white color wound turned into a fully hyperemic area due to the extravasation of the red blood cell of each group of rats.

After 30 minutes of burn induction, the respective gel was applied to each group of rats. On day 4, the HAM + AgNPs group's wound area expressed a wet crust while other rat groups expressed a slightly dry crust. On day 8, all groups treated with gel showed more dry crust compared to the negative control group. On day 12, detachment of crust edge was initiated, and HAM + AgNPs exposed much detachment of crust rather than other treatment groups. Scar tissue and contraction became visible in each treated group on day 16. On day 20, the wound area was minimized to a great extent. All rat groups treated with gels showed greater healing than the negative control group. However, the rat group treated with HAM + AgNPs gel expressed the best healing of wound area compared to other rat groups.

### 3.11. Wound Contraction Percentage

**Figure 6:**
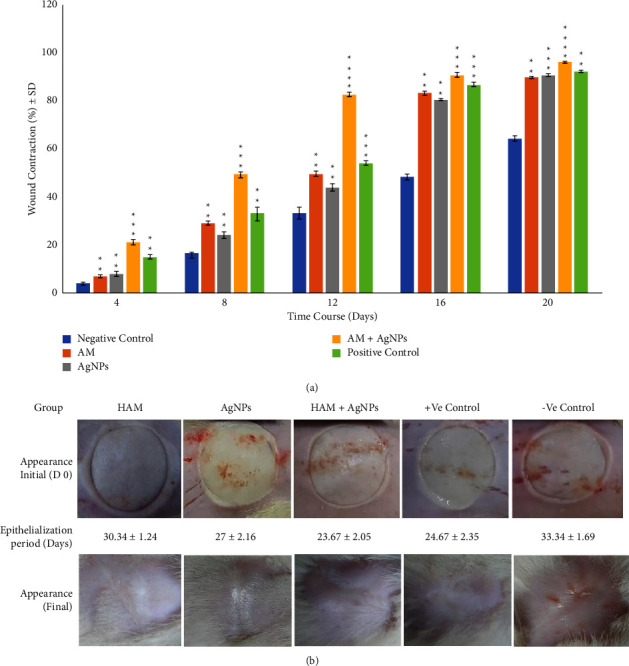
Wound contraction percentages and epithelialization periods. (a) Wound contraction percentages of different rat groups. The graph represents that different treatment groups have significantly better wound contraction percentages compared to the negative control (^*∗*^*p* ≤ 0.05, ^*∗∗*^*p* ≤ 0.01, ^*∗∗∗*^*p* ≤ 0.001, ^*∗∗∗∗*^*p* ≤ 0.0001). (b) Epithelialization periods of different rat groups; data were expressed as means ± SD.

For measuring the wound contraction, the wound area was defined as a percent for each time point. We observed an initial decrease in the wound area from the first 4 days and continued up to 20 days. On day 4, the wound contraction percentages were 7.07 ± 0.62 (HAM), 8.09 ± 0.95 (AgNPs), 15.11 ± 0.91 (+ve control), 3.95 ± 0.64 (-ve control), and 21.14 ± 1.5 (HAM + AgNPs) (Figure 6(a)). By day 20, negative control resulted in the least amount of wound closure with an average of 64.30 ± 1.18% contractions, and wounds treated with +ve control and HAM + AgNPs gel showed an average contraction of 92.37 ± 0.47% (*p* < 0.001) and 96.1 ± 0.27% (*p* < 0.0001), respectively. Wounds treated with HAM + AgNPs gel showed the best contraction percentage which is consistent with our observations throughout the study (Figure 6(a)).

### 3.12. Histopathology

**Figure 7:**
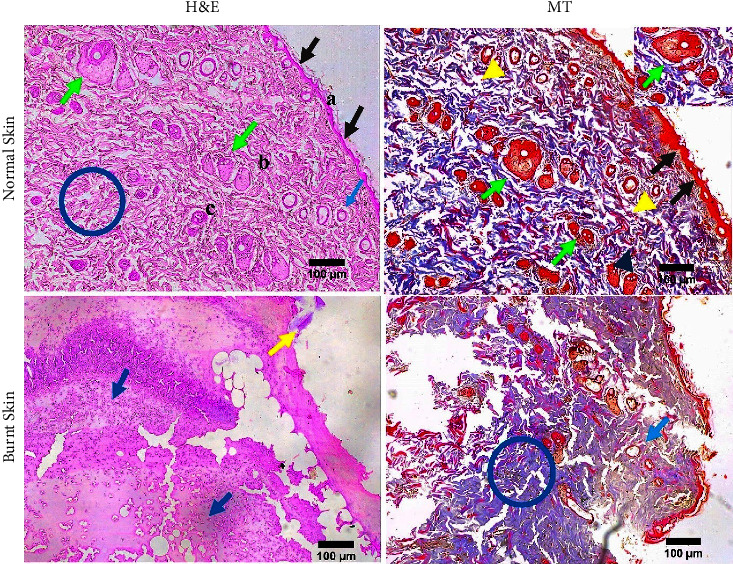
H&E and MT staining of normal skin and burnt skin. Blue arrow, black arrow, and yellow arrow indicate hair follicles, epithelial layer, and scab parts, respectively. Blue circle denotes collagen fibers; a, b, and c represent epidermis, dermis, and hypodermis, respectively. Dark blue arrow head and yellow arrow head indicate fibroblast cells and blood vessels, respectively. Dark blue arrow and green arrow highlight inflammatory cells and sebaceous glands, respectively (10x objective lens).

Normal skin consists of the epidermis, dermis, and hypodermis. Histopathological evaluation using H&E and MT staining showed that burning disrupts these layers and showed massive dissimilarities to the normal skin containing cells exudate and profound inflammatory mediators as well as inflammatory cells (Figure 7).

On day 7, wounds of all the groups had a large number of inflammatory cells, but the HAM + AgNPs treated group had less number than the others. On day 14, HAM + AgNPs and +Ve control groups had developed neogenetic papillary layers, neogenetic hair follicles, and blood vessels.

**Figure 8:**
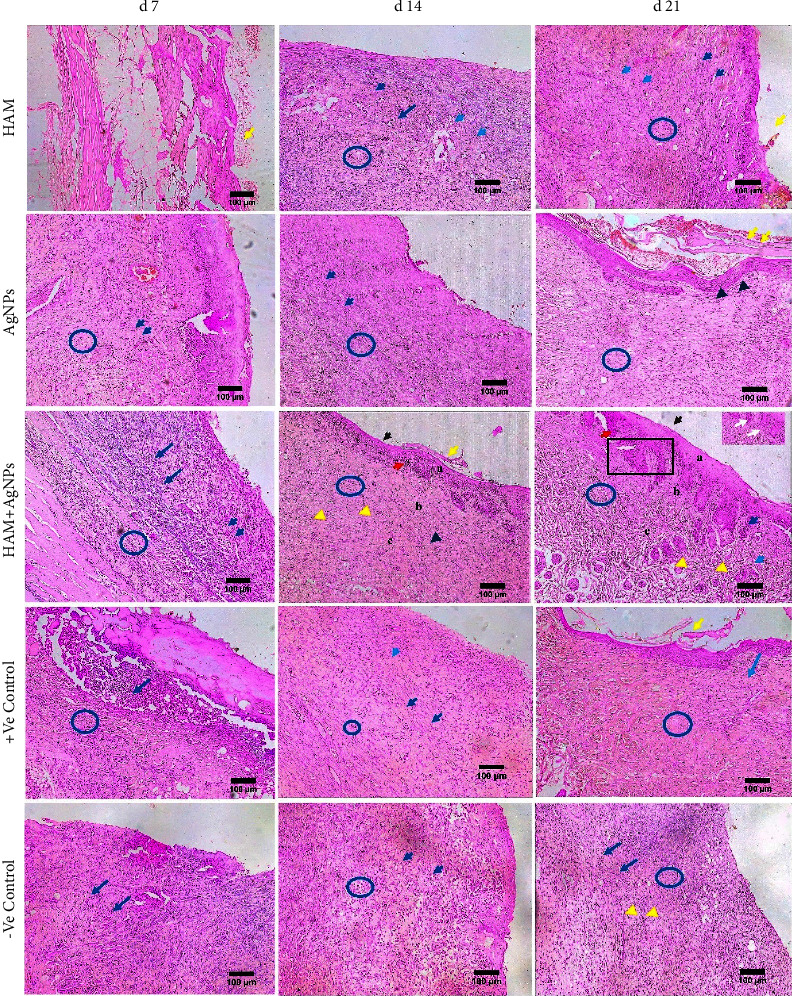
H&E staining. H&E staining of skin treated with HAM, AgNPs, HAM + AgNPs, +ve control, and −ve control. Blue arrow, black arrow, and red arrow project hair follicles, epithelial layer, and papillary dermis, respectively. Collagen fibers are denoted by blue circle; a, b, and c represent epidermis, dermis, and hypodermis, respectively. Yellow arrow, dark blue arrow, white arrow, and green arrow point to scab part, inflammatory cells, myofibroblasts, and sebaceous gland, respectively. Black arrow head, dark blue arrow head, yellow arrow head, and blue arrow head express keratinocytes, fibroblasts cells, blood vessels, and muscles, respectively (10x objective lens).

**Figure 9:**
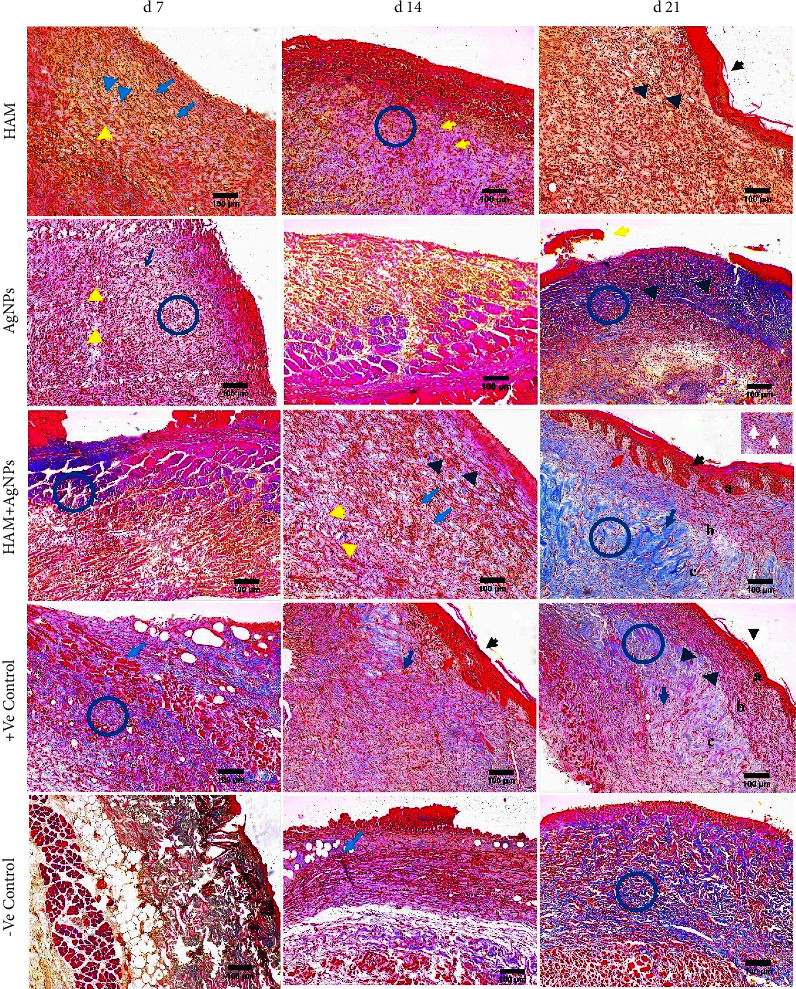
MT staining. MT staining of skin treated with HAM, AgNPs, HAM + AgNPs, +ve control, and −ve control. Blue arrow, black arrow, and red arrow indicate hair follicles, epithelial cells, and papillary dermis, respectively. Collagen fibers are denoted by blue circles. a, b, and c represent epidermis, dermis, and hypodermis, respectively. Yellow arrow, dark blue arrow, white arrow, and green arrow correspond to scab part, inflammatory cells, myofibroblasts, and sebaceous glands, respectively. Black arrow head, dark blue arrow head, and yellow arrow head mark to keratinocytes, fibroblast cells, and blood vessel, respectively (10x objective lens).

New blood vessels and hair follicles had developed slowly for other groups (Figures 8 and 9). On day 21, the newly generated part of the wounds treated with HAM + AgNPs became bigger, the scab part became smaller, the density of cells increased, and the ECM became comparable to normal skin, the whole epidermis was formed, and hypodermis grew to be more plentiful. ECM was poorly deposited in other groups, resulting in intact epithelium and dermis with no morphological alterations.

The migration of fibroblasts and keratinocytes, as well as extracellular matrix (ECM) molecules deposition to the wound site, was more prominent in the HAM + AgNPs group. In the case of–ve control, wound-healing events were delayed, resulting in wound contraction impairment. Thus, the HAM + AgNPs group exhibited superior wound healing compared to other groups.

### 3.13. Wound Epithelialization

Epithelialization was observed at each time point with visually defined epithelium with a matte layer different from the wound. Initially, all tissues had some epithelialization, and untreated showed the least wound epithelialization. Wounds treated with gels showed a significantly greater level (*p* < 0.01) of epithelialization than untreated and treated with HAM, and AgNPs demonstrated a final epithelialization of an average by day 30.34 ± 1.24 and 27 ± 2.16, respectively (Figure 6(b)). However, HAM + AgNPs treated wounds showed the most epithelialization (23.67 ± 2.05 days, *p* < 0.01) compared to other gel treated wounds (SI Figure 3).

## 4. Discussion

In this research, AgNPs incorporated HAM gel was assessed and compared preclinically with HAM and nanoparticle separately and with commercially available burn healing cream as a biological dressing for its healing effects. Here, HAM showed healing capabilities on burn wounds which had been used in burns treatment for decades [30, 31]. Koob et al. demonstrated that HAM was effective for wound healing and contained collagen (type IV, VII, XV, XVI, XVII, and XVIII); glycoproteins such as laminin (*α*3, *β*1, *β*2, *β*3, *γ*1, and *γ*2 chains), nidogen-1 and nidogen-2, fibronectin, fibulin-2, fibrillin-2; and proteoglycans such as perlecan and agrin play a vital role in wound healing [32]. A similar study showed that HAM acted as a basement membrane that facilitated the migration of epithelial cells, reinforced adhesion, promoted cell differentiation and maturation, and prevented cellular apoptosis as we found in our research [33]. A previous report by Gholipourmalekabadi et al. showed a 3D bilayered decellularized HAM/electro-spun silk fibroin membrane effectively accelerated the wound healing process, angiogenesis, and reepithelialization of hypertrophic scar in the rabbit ear model [34]. Here, Carbopol 934, a polymer of acrylic acid, was used in gel preparation for its hydrophilic, cross-linking, nontoxic, nonirritant, adhesive, absorbance enhancing, and biocompatible properties [35]. In a water solution at neutral pH, carbopol acts as an anionic polymer that contains carboxylate groups, i.e., many of the side chains of poly acrylic acid lose their protons and acquire a negative charge. The positively charged amino group of collagens and the negatively charged carboxylate of acrylic acid form a bond via free radical copolymerization [36, 37]. Besides, the hydrogen bond between the glycoprotein of the human amniotic membrane and the carboxylic acid of carbopol plays a significant role in the formation of a gel-like consistency [38]. A recent study by Chirayath et al. described the role of carbopol in gel preparation for its diverse beneficial properties, and Hayati et al. also showed the effect of carbopol in skin-burn wound gel similar to our study [39, 40].

AgNPs have gained much attention from researchers as AgNPs have several available reports about the anti-inflammatory, antiangiogenesis, and antibacterial effects which could promote burn wound healing [19, 41]. Due to the electrostatic interaction between positively charged silver ions and the negatively charged glycoprotein (carboxyl, phosphate, and amino groups) of the amniotic membrane, the formulated gel gained a consistent shape which is suitable for topical application [42]. Previous studies proved that AgNPs can inhibit bacterial growth and depress the activity of some membranous enzymes, which cause bacteria to die eventually by destroying bacterial membranous structure and permeability [43]. Our study also demonstrated the effect of AgNPs on burn healing which is consistent with many studies where AgNPs improved the rate of fibroblasts migration and helped to raise *α*-smooth muscle actin (producer of myofibroblasts), suggesting that AgNPs treated fibroblasts had the capability of converting into myofibroblasts; but in some studies, Pouraly et al. demonstrated that yet biologically produced AgNPs had possible dose-dependent toxic effects in the cell culture, produced AgNPs at their nontoxic doses had healing efficacy in the wound site [22, 44]. Moreover, Horue et al. investigated that bacterial cellulose (BC) containing 1% to 25% of montmorillonite (MMT) modified with silver was found to be biocompatible and nontoxic to mouse skin fibroblast, L929 cells after exposure for 12 h and 24 h [45]. Ag-doped bioactive glass is already used against extensively drug-resistant strains of these bacteria which were isolated from burn patients in an Ag concentration-dependent manner [46, 47]. It was also observed that the AgNPs were evaluated to be minimally cytotoxic, and there was no significant effect (*p* > 0.05) in terms of % viability of HeLa cell lines up to the concentrations of 120 *μ*g/ml [12]. Another study affirmed that silver had a potential toxic effect, such as argyria, an irreversible pigmentation of the skin and eyes due to inappropriate deposition of silver contradicts our research of using AgNPs [48]. In another study, a chitosan (CS) composite sponge dressing-load editurin-AgNPs showed no toxicity to all organs of mice with more effective inhibition of bacterial infection and promotion of wound healing processing by enhancement of reepithelialization as well as collagen formation [49].

Our findings were also similar to the above-mentioned studies. Here, HAM + AgNPs showed antibacterial activity against both gram +ve (*S. aureus*) and gram −ve (*E. coli, K. pneumoniae, P. aeruginosa*) bacteria (Figures 3(a), 3(b), and 4(a)) and had better-wound healing capabilities (Figures 5(b) and 6(a)). The ^1^H NMR(CDCl3) spectrum of HAM, AgNPs, and HAM + AgNPs gels presented the characteristic signals for HAM, carbopol 934, glycerol, and acrylic acid and contained chemical shift of different groups that resemble previous researches (Figure 2 and Supplementary Figure 2) [37, 50]. HAM + AgNPs promoted the contraction of the wound, accelerated the healing process, and reduced the epithelialization period (Figure 6(b)) better than other groups of rats. The wound areas of the HAM + AgNPs treated group were significantly (*p* < 0.0001) smaller than those of the control groups. H&E and MT staining showed that HAM + AgNPs treated wounds were healed faster in terms of neovascularization, reepithelialization, and the early presence of myofibroblast and keratinocytes, and cells and ECM density (Figures 7–9).

Therefore, our experiment exhibited that HAM + AgNPs gel was superior with respect to biocompatibility, percentage wound contraction (96.1 ± 0.27%), histopathologic observation, and epithelialization period (23 ± 2.05) assessments to commercially available burn creams, AgNPs, and HAM.

## 5. Conclusion

In the present study, HAM + AgNPs gel was applied to repair burn wounds in female Wistar rats in a preclinical trial. The gel was biocompatible and accelerated as well as strengthened wound healing. So, it can be an alternative dressing for burnt skin providing remarkable benefits by alleviating the pain sensation and enhancing the wound healing. Further studies and more investigations with larger sample sizes are required to use them clinically.

## Data Availability

Datasets used and/or analyzed during the current study are available from the corresponding author upon reasonable request.

## Abbreviations

HAM: Human amniotic membrane
AgNPs: Silver nanoparticles
H&E: Hematoxylin and eosin
MT: Masson's trichrome.

## Supplementary Materials

Supplementary Materials The detailed procedures of collection of human amniotic membrane (HAM), preparation of human amniotic membrane extract, synthesis of AgNPs, preparation of gel, characterization of formulated gel (determination of water absorption, swelling ratio, equilibrium water content, and swelling variation with pH), brine shrimp lethality test, physiological condition of rats, percentage of wound contraction, and epithelialization period have been mentioned in the supplementary information (SI) section.
