# Supplementary material for: Silver Nanoparticle Incorporated Human Amniotic Membrane Gel Accelerates Second-Degree Burn Wound Healing in Wister Rat
Source: Evid Based Complement Alternat Med. 2023 Apr 14;2023:9808556. doi: 10.1155/2023/9808556 (PMC10121346; doi:10.1155/2023/9808556)
Supplement: Supplementary Materials — The detailed procedures of collection of human amniotic membrane (HAM), preparation of human amniotic membrane extract, synthesis of AgNPs, preparation of gel, characterization of formulated gel (determination of water absorption, swelling ratio, equilibrium water content, and swelling variation with pH), brine shrimp lethality test, physiological condition of rats, percentage of wound contraction, and epithelialization period have been mentioned in the supplementary information (SI) section. [file 9808556.f1.docx]

**Title: Silver Nanoparticle Incorporated Human Amniotic Membrane Gel Accelerates Second-Degree Burn Wound Healing in Wister Rat**

**Supplementary Information files (SI)**

**Materials and methods:**

*Collection of Human Amniotic Membrane (HAM).* Human amniotic membranes were collected from seronegative (HIV, Hepatitis B and C viruses, and Syphilis) donors of cesarean deliveries (ages ranging from 25 to 35 years) from different maternity clinics in Dhaka. Subsequently, the samples were kept in a sterile container with sterile normal saline (0.9% NaCl, Sigma Aldrich, Germany). After that, samples were temporarily preserved in a refrigerator before transferring to the laboratory of the Institute of Tissue Banking and Biomaterial Research (ITBBR). Collected Human amniotic membranes were preserved in a freezer **(**RLHE0845, UK**)** below −20°C and used as per necessity.

*Preparation of Human Amniotic Membrane Extract*

a) Frozen HAM was thawed at room temperature and the amnion was separated from the chorion. Then, the separated amnion was washed in a 500ml beaker containing sterile saline.

b) After that, amnion was taken into a 250ml conical flask containing saline water and rotated at 160 rpm for 30 minutes on a shaker (KS501 digital, IKAWERKE, Japan).

c) After changing the saline water, step ‘b’ was repeated to clean the amnions properly. After cleaning, solution of penicillin and streptomycin (Oxoid Ltd. Ireland) were used to wash the amnions. Then, membranes were spread and oven dried (Salvis Lab, Swiss made). After that, membranes were frozen at subzero temperature and freeze-dried for 24 hours (Alpha1-4LD, CHRIST, Germany).

d) Then, the freeze-dried amnions were blended and HAM powder was irradiation at 25KGY. HAM Powder was then stored in the refrigerator for further use.

*Synthesis of AgNPs.* The AgNPs were prepared by the chemical reduction method where trisodium citrate dihydrate (C_6_H_5_O_7_Na_3_.2H_2_O) (SigmaAldrich.com, Germany) was used as a reducing agent as well as a stabilizing agent and Silver Nitrate (AgNO_3_) (Merck, Germany) was used as a precursor by following the procedure by Fang, Zhang, and Mu. All the ingredients used for the synthesis were prepared in distilled water. In this experiment, a conical flask containing 50 ml 0.001 M AgNO_3_ was heated to boil with continuous stirring. To this solution, 5 ml of 1% trisodium citrate dihydrate was added drop by drop using a burette.

The solution was mixed vigorously during this process and heated until the color changed to pale yellow. Then, the conical flask was removed from the heating instrument and stirred until room temperature was attained.

*Preparation of gel*

*Gel Formulation.* The best gel formulation with different carbopol 934 (Hi Media, India) concentrations (3, 4, and 5%) was selected. The finally selected gels were characterized based on several physical and biological properties. A total of nine types of gels were prepared using HAM, carbopol, glycerine (Merck, India), acrylic acid (Sigma Aldrich, Germany), silver nanoparticles, and triethanolamine (Hi Media, India). Gels were formulated by the following procedure:

*Solution A:* Firstly, a beaker was filled with a suitable amount of sterile distilled water. Then, accurately weighed carbopol 934 was gradually added and dispersed with the help of a magnetic stirrer.

*Solution B:* Accurate amount of HAM was dissolved and mixed under continuous stirring in another beaker with distilled water. HAM was homogenized using a homogenizer and an antimicrobial agent (acrylic acid) was added and mixed well. Then, glycerin was added slowly as a moisturizer under continuous stirring. After that, the silver nanoparticle was mixed with the solution.

Solution B was then put into a beaker containing solution A and mixed well. Finally, to neutralize the mixture, triethanolamine was added drop by drop, and mixing was continued until a translucent gel was created.

*Characterization of Formulated Gel:*

The formulated gels were characterized according to the following properties:

*Homogeneity.* All formulated gels were inspected visually for homogeneity and also tested if there were any aggregates.

*Spreadability.* Good spreadability is one of the optimal qualities for topical application. It indicates how easily the formulated gel can spreads on application area. 1 g of the gel was placed in a marked circle of 2 cm on a glass plate, which was then covered with second plate. A water filled beaker was kept on the upper glass plate for 5 minutes and the expansion in the radius was noted.

*pH measurement. The* pH of gel formulations was determined by using an electronic pH meter (HI-98107, Hanna Instruments, Italy). 2.5gm of the gel was dissolved in 25ml of distilled water and it was kept for 2 hrs. The pH of each gel was measured thrice and average values were calculated.

*Determination of water absorption and swelling ratio.* The gel samples were freeze-dried and weighed. Then the sample was immersed in distilled water and weighed again after removing excess water from the gel surface. Water absorption of the samples were computed as follows:

Water absorption [%] = [Wt – Wi] × 100; here, Wt is the weight of the swollen sample and Wi is the weight of dry samples.

For determining the swelling ratio, weighed freeze-dried gel samples were immersed in distilled water for 2 hours at room temperature. Then, the swollen gels were taken from distilled water and weighed after removing surface water. The swelling ratio was calculated as follows: Swelling ratio = [Ws – W_d_] / W_d,_ here, Ws is the weight of swollen gel and W_d_ is the weight of the dry gel.

*Determination of equilibrium water content and Swelling variation with pH.* The gel samples were freeze-dried and weighted. Then the samples were dipped in distilled water at room temperature and swelled until they gained a constant weight. Finally, the sample was weighed after removing surface water with filter paper.

The following formula was used to compute the Equilibrium water content (EWC): EWC [%] = [(Weq – Wi) / Weq] × 100; Where Weq is the weight of swollen gel at equilibrium and Wi is the initial weight of dry gel.

The gel samples were measured in buffer solutions with varied pH’s ranging from 3 to 8 at room temperature(25ºC) for 24 hrs in case of swelling ratio. The swelling ratio (S) was determined from the following equation: S = [(Wt – Wi) / Wi]; Where Wt is the weight of swollen gel after hydration for 24 hrs and Wi is the initial weight of dry gel.

*Brine shrimp lethality test.* Brine shrimp eggs collected locally were hatched in seawater(artificial) prepared from sea salt using Sodium chloride (NaCl), Potassium chloride (KCl), Calcium chloride (CaCl_2_.2H_2_O), Magnesium chloride (MgCl_2_.6H_2_O), and Magnesium sulfate (MgSO_4_.7H_2_0) (Sigma Aldrich, Germany).

*In Vivo study*

*Water and Food intake, and Body weight measurement of the rats.* Water and food intake, and body weight of the rats were taken every day during the experiment conducted.

*Percentage of wound contraction and* *Epithelialization period.* The area of wound was expressed as a percent to measure the wound contraction. From this, wound areas were calculated on respective days and the wound contraction percentage was calculated taking the initial size of the wound (491 mm^2^) as 100%.

Percentage Wound Contraction= [(Initial Wound Area – Final Wound Area) / Initial Wound Area] × 100.

The onset of epithelialization and the time (days) required for the epithelialization was noted.

**Results:**

*Physical Characteristics of Formulated Gels.* pH, swelling ratio, water absorption, swelling variation with pH, and equilibrium water content were determined to evaluate the physical characteristics of the gel.

*Physiological condition of rats.* The physiological condition of the rats (e.g., food and water intake, and body weight measurement) was noted (supplementary figure 1).


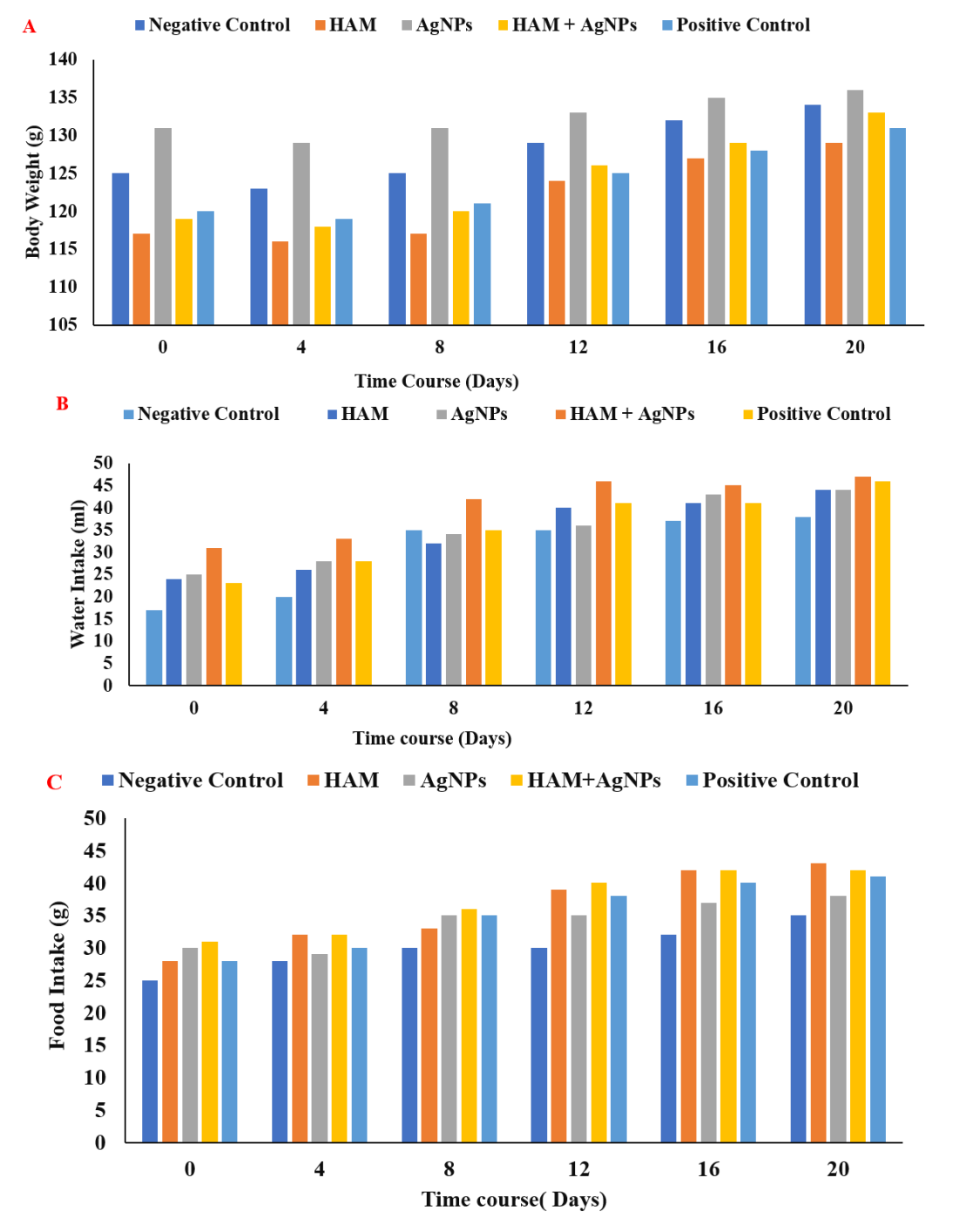


**Figure 1.** Average body weight, average water, and food intake of different rat groups. (A) Average Body weight, (B) Average Water Intake, (C) Average Food Intake.

*Nuclear magnetic resonance (NMR) spectroscopy.*

NMR spectroscopy of the gels (2% HAM and 5% Carbopol), (2% AgNPs and 5% Carbopol), and (2% HAM+AgNPs and 5% Carbopol) have been mentioned in supplementary Fig. 2.


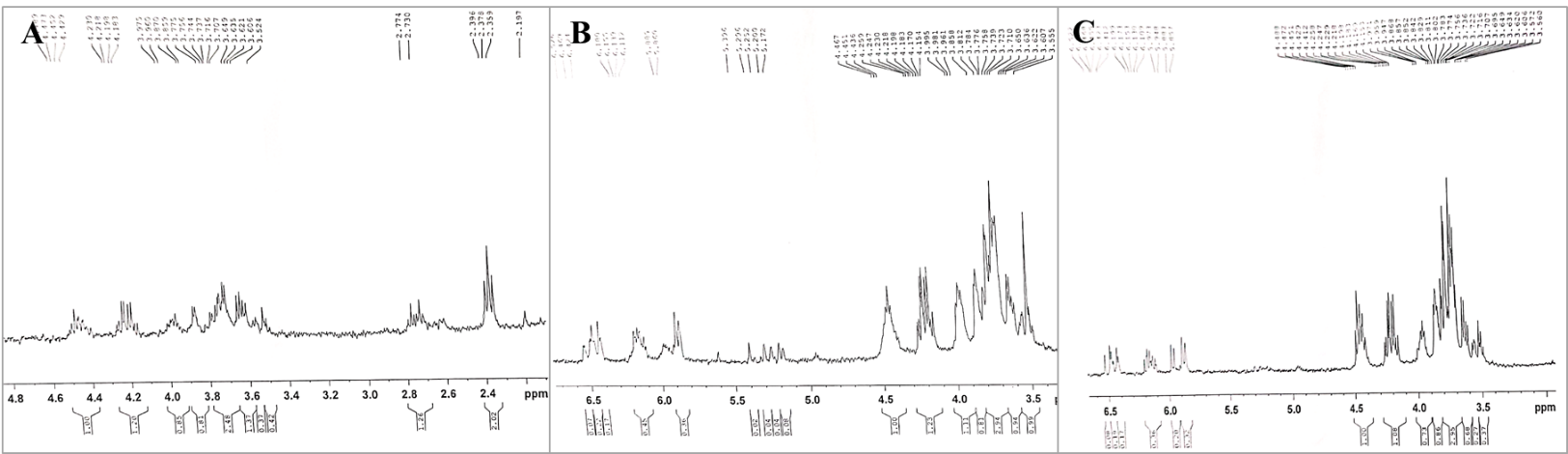


Fig. 2. NMR spectroscopy of the formulated gels. A- (2% HAM and 5% Carbopol), B- (2% AgNPs and 5% Carbopol), C-(2% HAM+AgNPs and 5% Carbopol).

*Epithelialization period.* The Epithelialization period of the rats was given in supplementary figure 3.


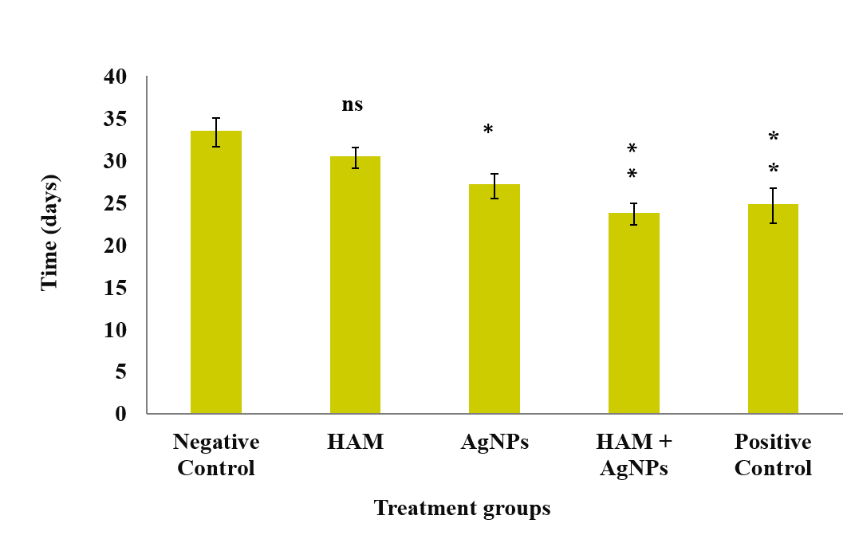


Figure 3. Epithelialization periods of different rat groups. The graph represents significant changes in the mean value of epithelization periods of treatment groups compared to the negative control (*p < 0.05, **p < 0.01, ns: nonsignificant)
